# Is the neglected tropical disease mass drug administration campaign approach an effective strategy to deliver universal health coverage? A case study of the Liberia neglected tropical disease programme

**DOI:** 10.1093/inthealth/ihad035

**Published:** 2023-05-16

**Authors:** Andrew Tate, Karsor Kollie, Laura Senyonjo, Hugh Sturrock, Phil Downs, Simon Bush, Alex Bedell, David Molyneux

**Affiliations:** Sightsavers, 35 Perrymount Road, Haywards Heath, Sussex, RH16 3BW, UK; Director, Programme for Neglected Tropical Diseases, Monrovia, Liberia; Sightsavers, 35 Perrymount Road, Haywards Heath, Sussex, RH16 3BW, UK; Locational Analysis Ltd, Poole, Dorset BH16 6FA, UK; Sightsavers, 35 Perrymount Road, Haywards Heath, Sussex, RH16 3BW, UK; Director, Neglected Tropical Diseases, Sightsavers, P.O. Box KIA 18190, Airport, Accra, Ghana; Liberia Country Office, Sightsavers, Monrovia, Liberia; Department of Tropical Disease Biology, Liverpool School of Tropical Medicine, Pembroke Place, Liverpool L3 5QA, UK

**Keywords:** geospatial modelling, Liberia, mass drug administration, neglected tropical diseases, universal health coverage

## Abstract

**Background:**

Access to affordable, quality healthcare is the key element of universal health coverage (UHC). This study examines the effectiveness of the neglected tropical disease (NTD) mass drug administration (MDA) campaign approach as a means to deliver UHC, using the example of the Liberia national programme.

**Methods:**

We first mapped the location of 3195 communities from the 2019 national MDA treatment data reporting record of Liberia. The association between coverage for onchocerciasis and lymphatic filariasis treatment achieved in these communities was then explored using a binomial geo-additive model. This model employed three key determinants for community ‘remoteness’: population density and the modelled travel time of communities to their supporting health facility and to their nearest major settlement.

**Results:**

Maps produced highlight a small number of clusters of low treatment coverage in Liberia. Statistical analysis suggests there is a complex relationship between treatment coverage and geographic location.

**Conclusions:**

We accept the MDA campaign approach is a valid mechanism to reach geographically marginal communities and, as such, has the potential to deliver UHC. We recognise there are specific limitations requiring further study.

## Introduction

The World Health Organization (WHO) estimates that at least half of the global population does not receive the health services they need. About 100 million people are pushed into extreme poverty through catastrophic health expenditures each year because of out-of-pocket spending on health, with some 930 million people spending >10% of their income on healthcare.^1^

The WHO defines universal health coverage (UHC) as ‘that all people have access to the health services they need, when and where they need them, without financial hardship. It includes the full range of essential health services, from health promotion to prevention, treatment, rehabilitation, and palliative care’.^1,2^

UHC should be based on effective, people-centred primary healthcare. Strong health systems are rooted in the communities they serve and focus not only on preventing and treating disease and illness, but also on helping to improve well-being and quality of life. However, translating these aspirations into reality provides a challenge for all health providers and policymakers. While global performance as per the UHC effective coverage index has improved,^3^ a recent United Nations (UN) report on progress towards the UN Sustainable Development Goal (SDG) targets recognises ‘[t]he world is falling short on its promise of universal health coverage by 2030’, a key element of the health targets lately exacerbated by the impact of coronavirus disease 2019 (COVID-19).^4^

In the context of neglected tropical diseases (NTDs), a key strategy for the implementation of control and elimination programmes is preventive chemotherapy (PC). This strategy is based on annual or biannual treatment campaigns with donated drugs via mass drug administration (MDA) for a group of five conditions: onchocerciasis, lymphatic filariasis (LF), schistosomiasis, soil-transmitted helminthiases and trachoma.^5^ A WHO and World Bank report^2^ on monitoring of UHC is explicit that ‘monitoring preventive chemotherapy remains key to ensuring that the diseases of the least well-off are being prioritised from the very beginning of the path to UHC’. The Third Report of the WHO on Neglected Tropical Diseases used the term ‘litmus test’ to suggest that effective control of NTDs will be a measure of success in achieving the health targets of the UN SDGs.^6–8^ Allied to the concept of the ‘litmus test’ is the ethos of ‘leave no one behind’, reflecting the need to ensure that the concept of equity prevails in consideration of reaching the least-accessible, deprived, marginalised, vulnerable and hard-to-reach communities and individuals.

It is widely recognised that NTD interventions to control or eliminate diseases amenable to PC have achieved remarkable success in delivering donated drugs over at least 2 decades. Initial community-based programmes commenced in the late 1980s following the donation of Mectizan (brand name for ivermectin [IVM]) by Merck & Co. ‘for as long as needed’ to control or eliminate onchocerciasis. From this, the concept of community directed treatment with ivermectin (CDTI) was shown to be an effective, resilient and sustainable approach to engaging and motivating communities, ensuring high treatment coverage compatible with an impact on morbidity.^9^ Initiated in 1995, the African Programme for Onchocerciasis Control (APOC) adopted CDTI as a core strategy and expanded distribution of Mectizan through annual treatments to some 19 countries in Africa.^10^ In the Americas, the Onchocerciasis Elimination Programme for the Americas (OEPA) twice-yearly Mectizan treatment has eliminated transmission of onchocerciasis in four countries: Colombia, Ecuador, Guatemala and Mexico.^11^

Over the 5 y prior to the COVID-19 pandemic, the WHO reported that each year some 1 billion treatments for PC NTDs have been distributed in >70 countries.^12^ This remarkable achievement is supported by the efforts of countless volunteer community-directed distributors (CDDs), particularly in Africa, the unsung ‘foot soldiers’ in the MDA campaign approach.^13^ A review of the Mectizan Donation Programme (MDP)^14^ showed that in any one year, some 70% of the IVM approved for treatment by the MDP and WHO reached eligible communities within 12 months. Given that onchocerciasis is a predominantly rural disease, this sustained achievement of drug delivery to populations often distant from formal health facilities emphasises the robustness of community-led activities.

Our research question was does MDA through the CDTI delivery platform reach populations hitherto beyond the reach of the formal health services? This study was then developed to achieve a measured understanding of the effectiveness of the MDA campaign approach to achieve geographic equity and, as such, its value to support efforts for UHC. As a country example, we used Liberia (47.6 UHC effective coverage index^3^), and leveraged replicable geospatial modelling techniques to explore national MDA campaign data for the delivery of IVM and albendazole (ALB) for the treatment of onchocerciasis and LF.

## Methods

### Background to drug treatment in Liberia

**Figure 1.**
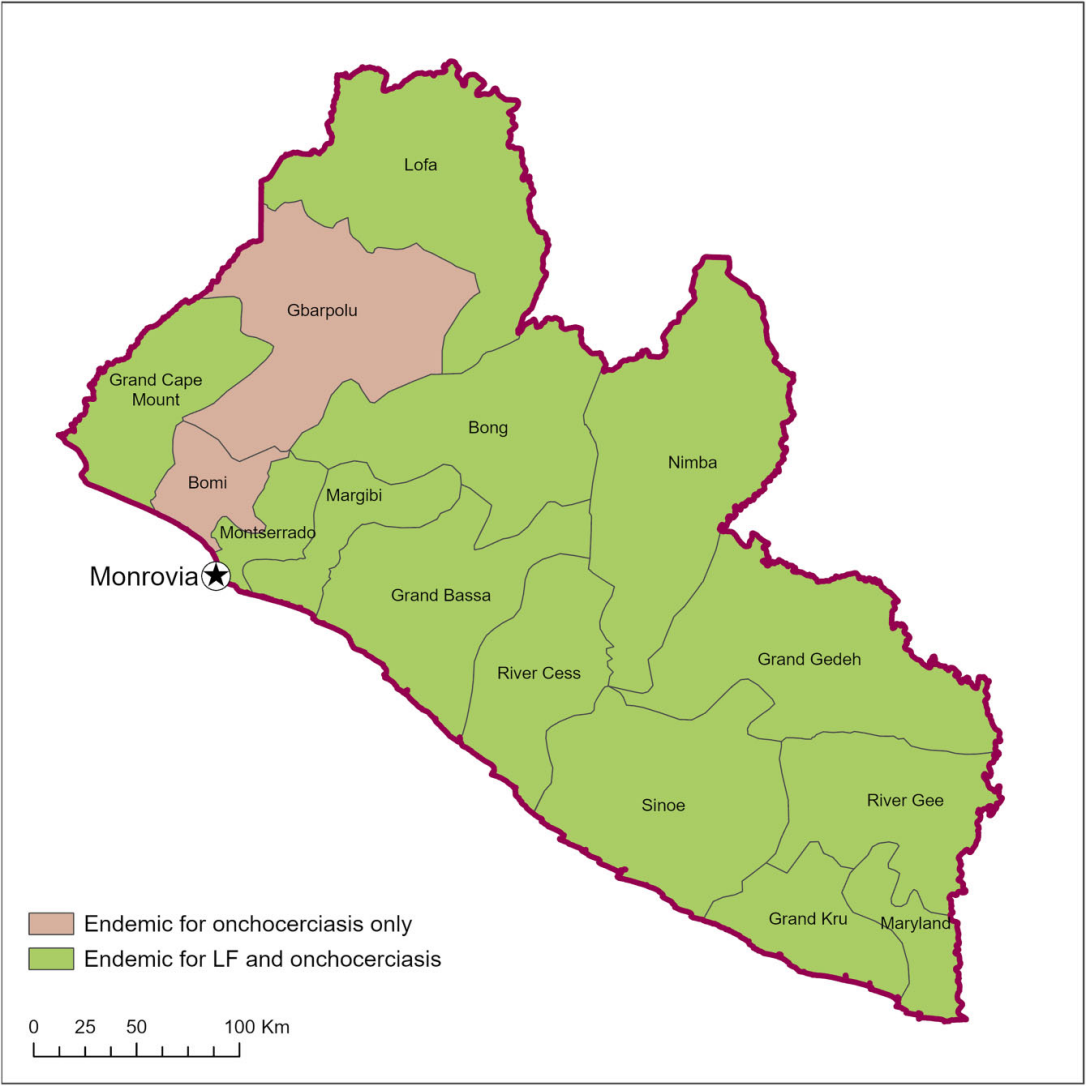
Endemicity of onchocerciasis and LF in Liberia.

Onchocerciasis is endemic to all 15 counties of Liberia and LF is endemic to 13 (Figure 1). CDTI for onchocerciasis started in 2000, following rapid epidemiological mapping of onchocerciasis (REMO) in 1999. Subsequent MDA campaigns were impacted by civil war until 2003, and population treatment coverage remained low until 2010. Drug treatment for LF started in 2012.^15^ MDA campaigns are undertaken once a year, with drug distribution per community coordinated by front-line health facilities (FLHFs). These FLHFs dispense drugs to CDDs for house-to-house distribution supported by district, county and national supervisors.

During an MDA campaign, comprehensive treatment registers are compiled by CDDs. These include a count of the number of eligible people given IVM and ALB and their sex and age. Subsequently, individual community treatment registers are aggregated at the serving FLHF, then at the district, county and national levels. Drug coverage for each listed community (community MDA distribution point) is calculated as the total number of individuals who received drugs over the total population enumerated by the CDD at the time of distribution. Total population is inclusive of those ineligible for treatment, including children <5 y of age, pregnant women, breastfeeding mothers and the severely ill.

Of an estimated national population of 4.9 million^16^ in 2019, 2 575 727 people were reported in the 2019 national MDA treatment record as being treated for onchocerciasis and 2 408 746 for LF. The national population here is inclusive of the capital, Monrovia (estimated population 1 467 000^17^), which is itself not targeted for treatment, and those otherwise ineligible. It is important to note that this study used preliminary national data. A final consolidation prior to reporting to the WHO corrected for an additional 4347 people treated in Gbarpolu, Maryland and Sinoe Counties (+0.2% adjustment). Nationally, 11/15 treated counties achieved aggregate WHO-recommended minimum epidemiologic population treatment coverage of 65% for onchocerciasis and 9/13 treated counties for LF.

### Geospatial modelling using Liberia MDA campaign data

The coordinates of community MDA distribution points^18^ and supporting FLHFs^19^ were matched from publicly accessible data sourced from the Humanitarian Data Exchange (HDX) to those listed in the national MDA treatment record of Liberia using the Excel fuzzy lookup add-in (Microsoft, Redmond, WA, USA). This was followed by a process of manual identification.

Three variables to act as determinants for community ‘remoteness’ were then selected for exploration in the geospatial model. These were population density and the travel time of mapped community MDA distribution points to their supporting FLHF and the nearest major settlement.^20^ The latter here are 45 settlements in Liberia designated as ‘city’ or ‘town’ only.

**Figure 2.**
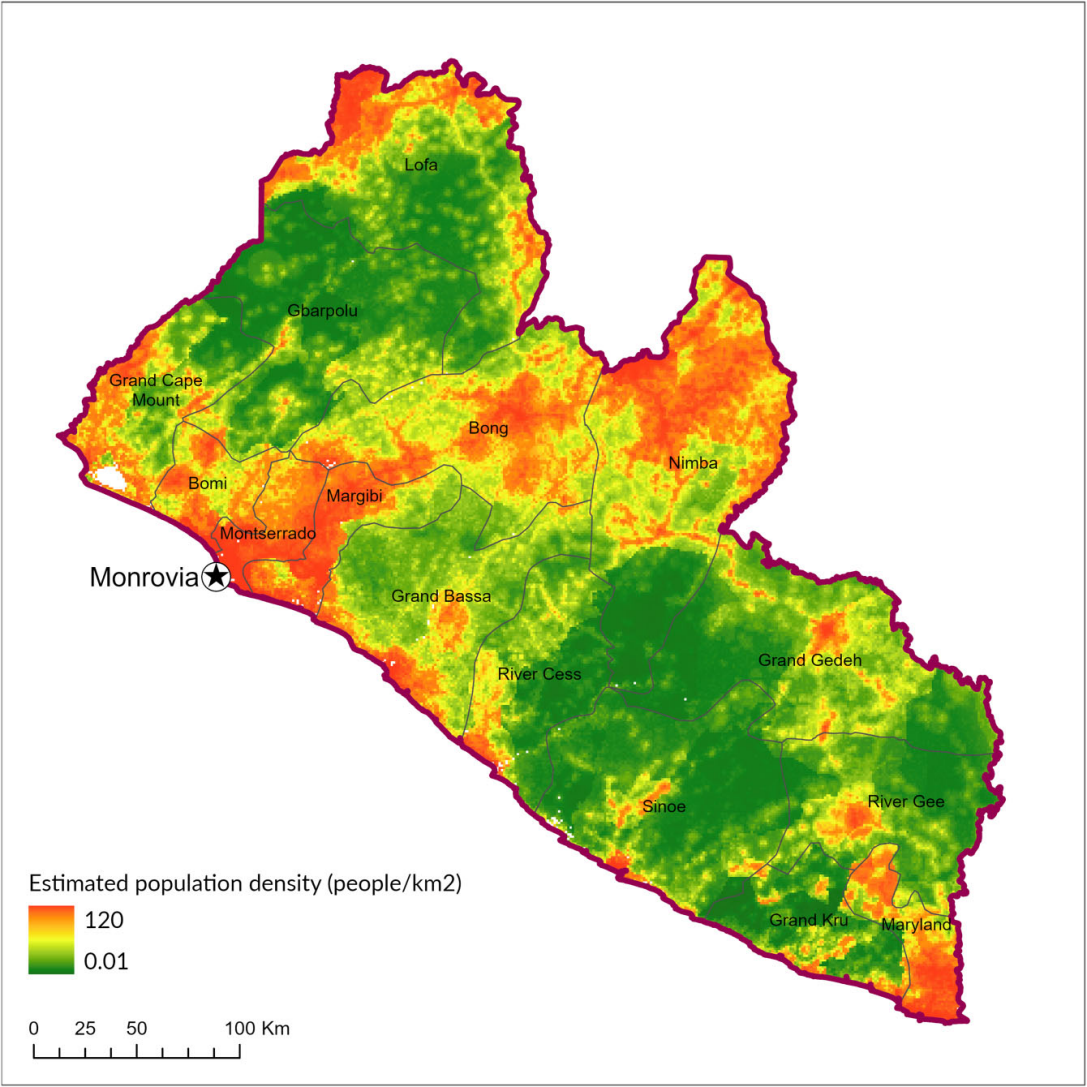
Population density in Liberia.^21^

While a raster file for population density at a resolution of 3 arcsec (approximately 1 km at the equator) and adjusted to match corresponding UN population estimates was sourced from WorldPop^21^ (see Figure 2), the calculation of travel time from community MDA distribution points to the nearest FLHF and to the nearest major settlement first required construction of a cost surface. Also known as a friction surface, this is a gridded surface for which each cell is assigned an associated cost relative to the level of effort to travel through it from a defined origin point.

Construction of the cost surface and the subsequent modelling of travel time in minutes from community MDA distribution points to FLHFs and major settlements was undertaken using the WHO-developed open-source GIS extension AccessMod.^22,23^ Note that the use of AccessMod does not here imply endorsement by the WHO to our study. The input datasets used are listed in Table 1, noting that the spatial resolution of input raster files was first resampled in ArcGIS Pro (Esri, Redlands, CA, USA) to a standardised 1-km resolution.

**Table 1.** Input datasets used to model travel time in AccessMod

	Land use	Road network	Hydrography	Digital elevation model	FLHF	Major settlements
Format	Raster	Vector	Vector	Raster	Vector	Vector
Year	2019	2018	2021	2018	2014	2021
Source	Sentinel-2^24^	OCHA^25^	HOT^26^	CGIARCSI^27^	Standby Task Force^19^	HOT^20^
Spatial resolution	10 m	N/A	N/A	30 m	N/A	N/A
Purpose	To account for travel speed by land cover, i.e. urban or forested	To account for travel speed by road network and road surface	To account for barriers to movement, i.e. rivers or lakes	To adjust for elevation and slope when mode of travel is walking	Location towards which travel speed is calculated	Location towards which travel speed is calculated

Before the model could be run, it was necessary to assign a travel speed in km/h to each land cover type in the cost surface according to the assumed mode of travel. Where the assumed mode of travel was walking, AccessMod corrected for speed as a function of slope as per Tobler's hiking function.^28^ Travel speeds were allocated to land cover types as outlined in Table 2, assuming the use of motorised transport on major and secondary roads, and walking otherwise. Speeds in km/h were allocated as per the units employed by Chen et al.^29^ in their 2017 study of emergency obstetric and neonatal care in the Kigoma region of Tanzania.

**Table 2.** Allocated travel speed per land cover type

Land cover type	Travel scenario	Travel speed (km/h)
Forest	Walking	1.0
Cropland	Walking	1.7
Grassland	Walking	1.7
Scrubland	Walking	1.7
Urban	Walking	2.5
Major road	Motorised transport	50
Secondary road	Motorised transport	40
Local road (track)	Walking	2.5

The output raster files generated by AccessMod for both travel time from communities to their nearest FLHF and major settlement were then imported into ArcGIS Pro and combined with the raster file for population density into a single authoritative dataset. Values of each of these three raster layers were extracted at each point for which coverage data were available (i.e. each geo-referenced community MDA distribution point). Associations between the explanatory variables and coverage were then explored using a binomial geo-additive model. A geo-additive model is a generalised additive model^30^ that includes a smooth spatial term to model residual spatial effects not explained by the covariates. The geo-additive model fit non-linear relationships with each covariate using penalized splines with a bivariate Gaussian process smooth on latitude and longitude to allow for an additional spatial effect. An additional penalty term was added to allow unimportant covariates to essentially be selected out of the model.^31^ The Akaike information criterion was used to determine the backwards stepwise inclusion of covariates. This model was used to generate maps of predicted population treatment coverage. In addition, probability distributions allowed estimation of an ‘exceedance probability’, the probability that coverage is above a certain threshold. In this case, the probability that coverage is above the WHO-recommended epidemiologic population treatment coverage threshold of 65% was estimated. Both the predicted coverage and the probability that coverage was >65% were made at 1-km resolution.^32^

## Results

**Figure 3.**
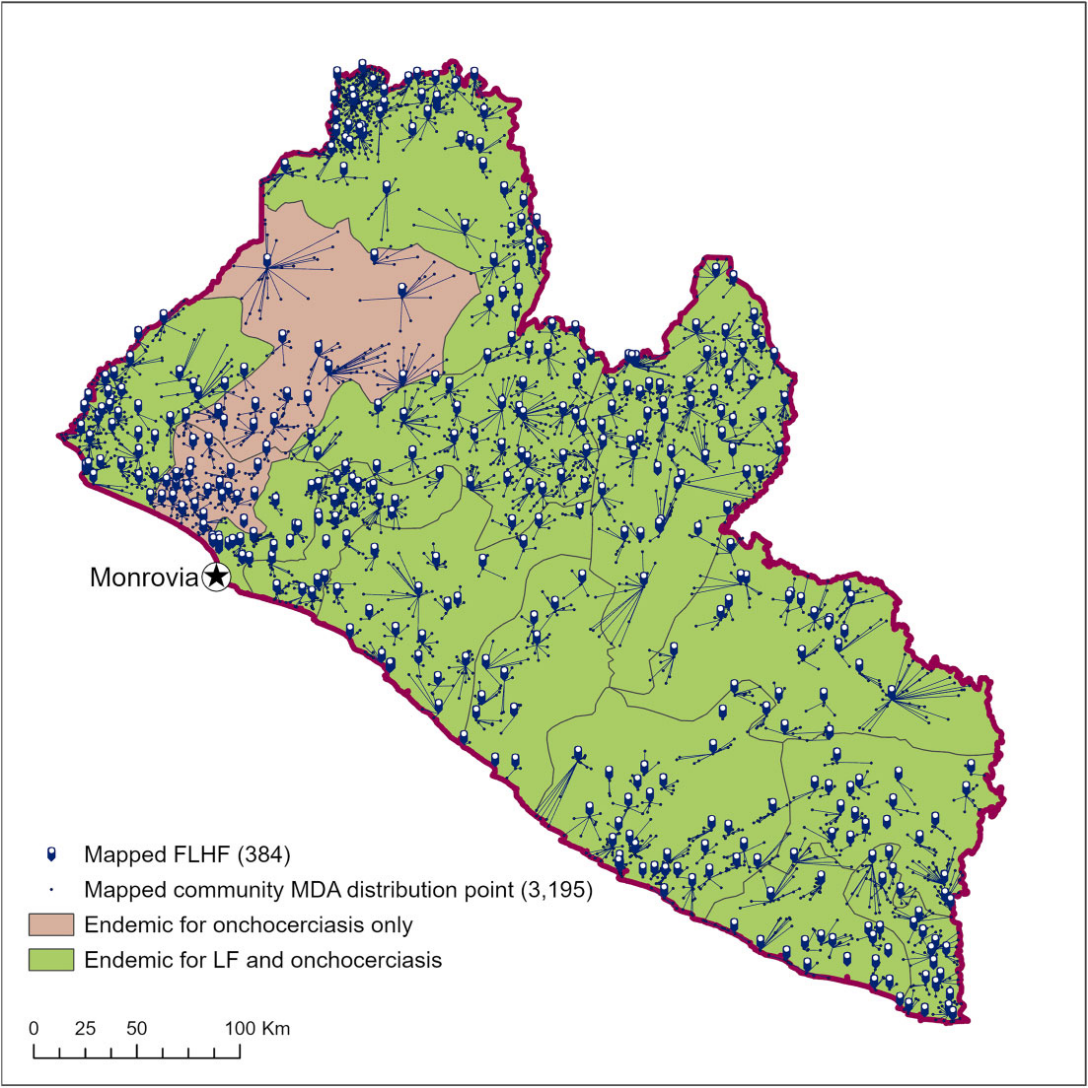
Results of mapping from the 2019 national MDA treatment record in Liberia.

From the 2019 national MDA treatment record, a total of 3195 community MDA distribution points of 5095 listed (63%) and 384 FLHFs of 478 listed (80%) were successfully matched and mapped. Any communities that could not be identified or for which the location of the associated FLHF could not be identified were removed from the analysis (1900 community MDA distribution points and 94 FLHFs). While our most complete data were in western Liberia, where 1168 communities were mapped in Bomi, Grand Cape Mount, Gbarpolu and Lofa Counties (79.5% of all listed community MDA distribution points in these counties), geolocation was a particular issue in more urban and peri-urban areas (excepting that Monrovia is not targeted for treatment). This challenge was due to many differences in the names of community MDA distribution points in national reporting and the names of communities available through publicly accessible data sources. Figure 3 illustrates the results of this initial process, wherein geolocated communities have been linked by line to their supporting FLHF. In effect, this represents each individual mapped FLHF treatment catchment area.

**Figure 4.**
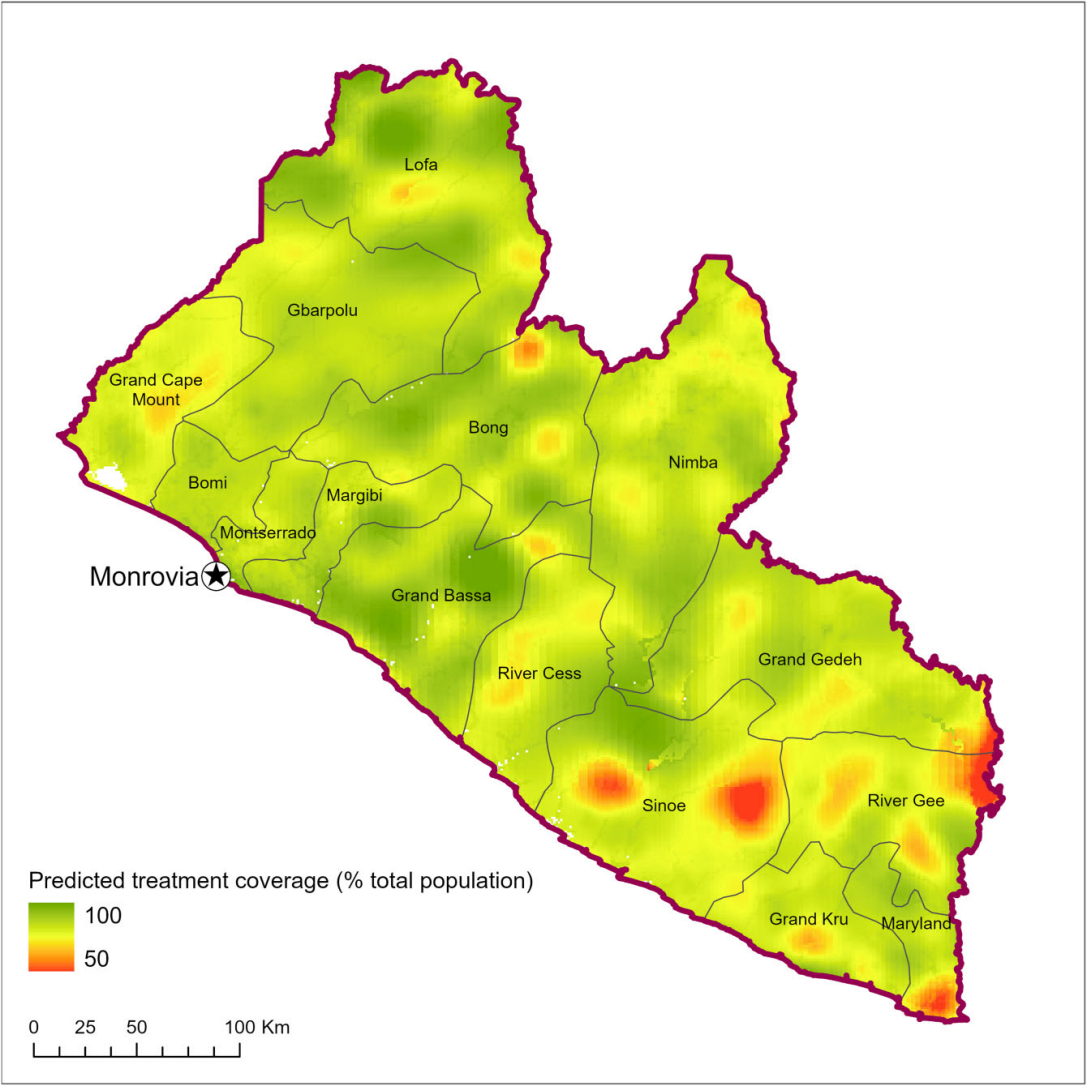
Mapped predicted population treatment coverage in Liberia.

**Figure 5.**
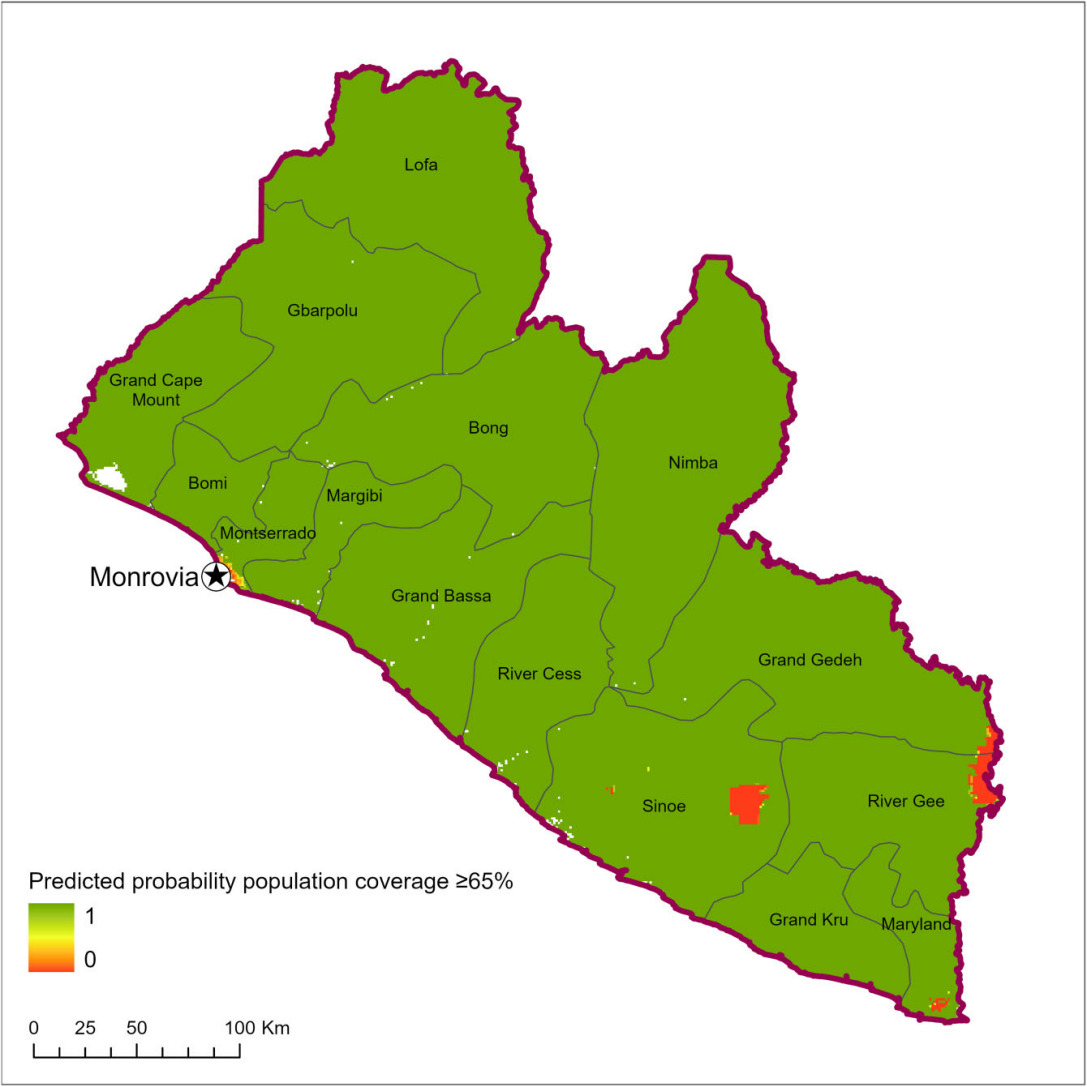
Mapped exceeded probability (≥65%) in Liberia.

The results of the geospatial modelling are shown in Figures 4 and 5. Figure 4 shows a number of pockets of lower observed treatment coverage in Liberia. However, when focusing on just those areas where it is probable that coverage will be below the minimum epidemiologic population treatment coverage of 65% (Figure 5), specific clusters of concern are evident adjacent to Sapo National Park in Sinoe County and east of the Dube River in River Gee County.

**Figure 6.**
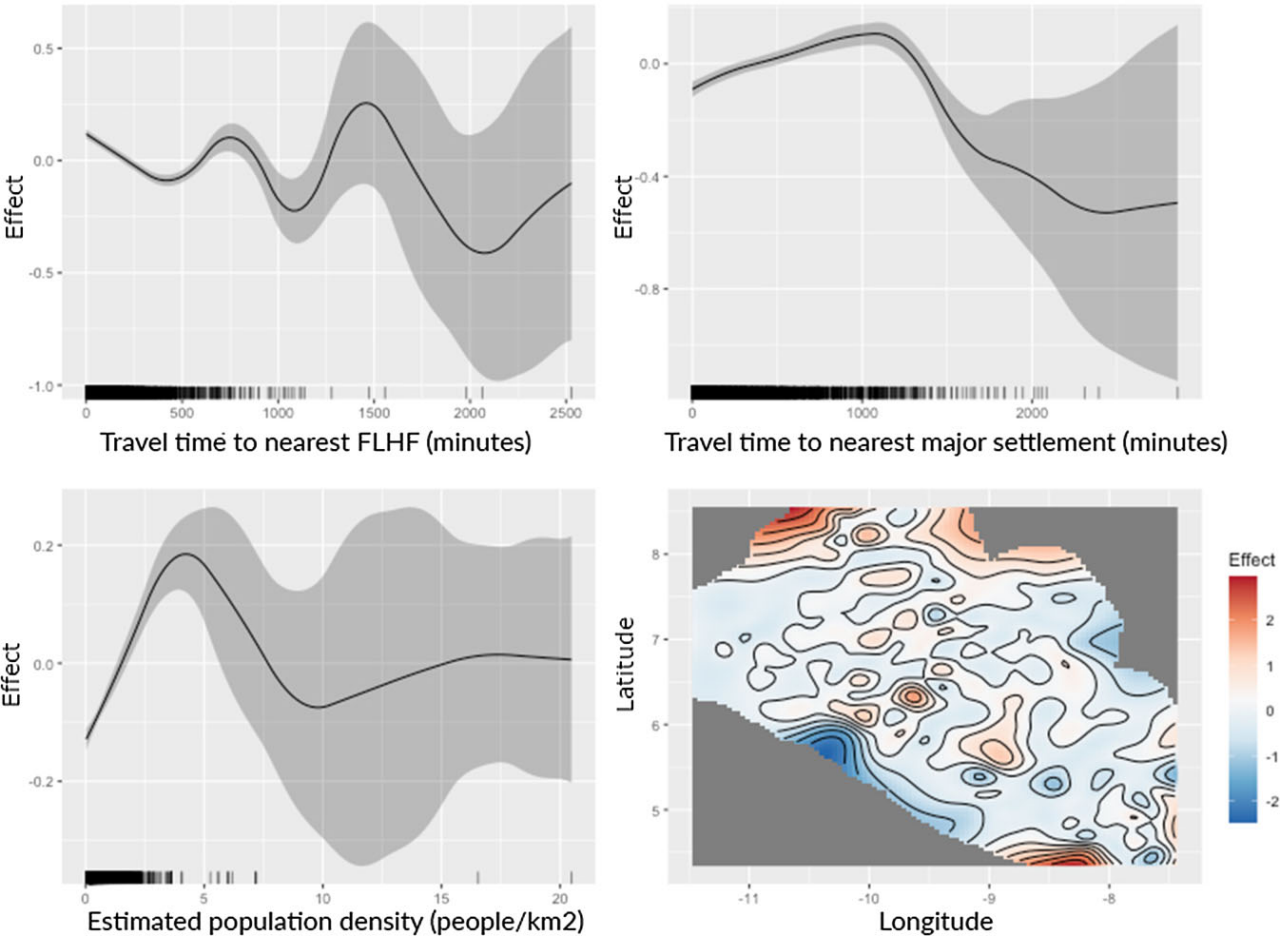
Relationships between explanatory variables and coverage as estimated by generalized additive modelling.

Figure 6 suggests that all three explanatory variables show non-linear relationships with treatment coverage. For travel time to the nearest FLHF, there appears to be a complex non-linear relationship, with a general trend of those living further from facilities having slightly lower coverage. In contrast, travel time to the nearest major settlement appears to have a positive relationship with coverage up to around 20 h. Those living further than this have increasingly lower coverage. Note that our model—as per Table 2—is inclusive of vehicular travel on major and secondary roads only, with travel otherwise on foot. Of the mapped communities, 95% were <20 h from their nearest major settlement. For population density, while the model outputs suggest a non-linear relationship, the vast majority (98%) of observations were from areas with a population density of 0.03–5 people/km^2^. Within this range, modelling suggests a relatively linear positive relationship, with higher-density areas having higher coverage. Plots of the spatial effect suggest a residual spatial effect, suggesting there are factors affecting coverage beyond those examined here.

## Discussion

This study was driven by the need to understand if the MDA campaign approach as a model presents an effective approach for supporting efforts for UHC. In the context of Liberia, our analysed data support the assertion that the MDA campaign approach is a valid mechanism to reach geographically marginal communities and that the MDA campaign approach is therefore a valuable tool with potential for delivering UHC. However, we also recognise that there is a complex relationship between treatment coverage and geographic location.

While there are few studies that provide a geospatial analysis of the reach of key health interventions at the community level, our conclusion of MDA campaign effectiveness compares favourably with the coverage of long-lasting insecticide-treated nets (LLINs) delivered through campaigns in Burkina Faso. Here, coverage was shown to decrease in populations >5 km from a health facility (odds ratio 0.57 [95% confidence interval 0.42 to 0.76]).^33^ Comparing the assertion of 70% appropriate MDA campaign drug use by Hodgkin and Jennisken^14^ with a 1994 study by the World Bank^34^ (reporting only 12% of drugs entering a health system are appropriately used) further highlights the value of the MDA campaign approach, notwithstanding also its inherent flexibility. This is evident by the fact that the MDA campaign approach, including CDDs, has been successfully repurposed for alternative health interventions in recent years, including those aimed at reducing infant mortality^35^ and pandemic responses.^36^

This study has specific limitations that present clear opportunities for future investigation.

First, Liberia is a relatively small country with a higher number of health facilities compared with other post-conflict low- and middle-income countries. It would be beneficial to conduct a multicountry analysis including larger countries with greater distances between health facilities or from major settlements to see if coverage remains equitable over larger distances and in different contexts and settings.

Second, this study is constrained by an incomplete sample of national reporting due to the difficulties of matching community names in more urban and peri-urban areas. To improve the validity of the model in Liberia, efforts will be made to confirm the location of identified community MDA distribution points and FLHFs and to locate those missing from our sample. We will also run the model with subsequent years of treatment data, which will further serve to verify if there are communities missed or inconsistently treated. As our challenges with geolocation in more urban and peri-urban areas are broadly illustrative of the challenges MDA programmes have in these settings,^37^ it may be helpful to consider removing more urbanised areas from future studies. However, it should be noted that the African Development Bank projects that by 2030, 350 million people in Africa will live in cities (some 50% of the total population) as a result of rural–urban migration trends.^38^

Finally, it is widely recognised that there are issues with the quality of data recorded in CDD treatment registers and population estimates. This is a broad concern across all MDA treatment programmes and one must therefore consider this constraint for any study that uses MDA campaign data. Post-MDA surveys where coverage (interview) data are collected from a cluster random sample of the population is an alternative way to obtain a coverage estimate, and efforts should be made to compare the results of these studies as conducted in Liberia with our analysis. Such an approach, however, not only requires more resources, but can also suffer from issues such as recall and sampling bias.

## Conclusions

While requiring further investigation, specifically to compare our approach across multiple years of treatment data in Liberia and to apply the same methodology elsewhere, this study has presented an argument for the potential of the MDA campaign approach in achieving UHC. Our geospatial method may be easily replicated (although requiring a level of Geographic Information System expertise to operate) and adapted to model population coverage of any community-based intervention. Further, the modelled output maps generated may be used by a national programme to inform the allocation of resources to targeted initiatives in identified areas of treatment coverage concern, such as supervisor and CDD training, and social mobilisation activities. Without this approach, validating the aspirations of UHC in the context of the remotest and most disadvantaged communities will remain a significant challenge.

## Data Availability

Treatment data for Liberia at a county level is publicly available for download from the WHO ESPEN portal (https://espen.afro.who.int/). Permission is required from the Liberian Ministry of Health to access MDA treatment data at the community level.

## References

[1] World Health Organization . Universal Health Coverage. Available from: https://www.who.int/health-topics/universal-health-coverage#tab=tab_1 [accessed 15 December 2021].

[2] World Health Organization, World Bank . Tracking universal health coverage: the first global monitoring report. Geneva: World Health Organization; 2015. Available from: https://www.who.int/publications/i/item/9789241564977

[3] GBD 2019 Universal Health Coverage Collaborators . Measuring universal health coverage based on an index of effective coverage of health services in 204 countries and territories, 1990–2019: a systematic analysis for the Global Burden of Disease Study 2019. Lancet. 2020;396(10258):1250–84. 32861314 10.1016/S0140-6736(20)30750-9 PMC7562819

[4] United Nations . The Sustainable Development Goals Report 2020. New York: United Nations; 2020. Available from: https://unstats.un.org/sdgs/report/2020/ [accessed 29 April 2023].

[5] Casulli A. New global targets for NTDs in the WHO roadmap 2021–2030. PLoS Negl Trop Dis. 2001;15(5):e0009373. 10.1371/journal.pntd.0009373 PMC8118239 33983940

[6] World Health Organization . Investing to overcome the global impact of neglected tropical diseases: third WHO report on neglected tropical diseases 2015. Geneva: World Health Organization; 2015. Available from: https://apps.who.int/iris/handle/10665/152781 [accessed 15 December 2021].

[7] Bangert M, Molyneux DH, Lindsay SW et al. The cross-cutting contribution of the end of neglected tropical diseases to the sustainable development goals. Infect Dis Poverty. 2017;6:73. 28372566 10.1186/s40249-017-0288-0 PMC5379574

[8] Dean L, Ozano K, Adekeye O et al. Neglected tropical diseases as a ‘litmus test’ for universal health coverage? Understanding who is left behind and why in mass drug administration: lessons from four country contexts. PLoS Negl Trop Dis. 2019;13(11):e0007847. 31751336 10.1371/journal.pntd.0007847 PMC6871774

[9] Meredith SEO, Cross C, Amazigo UV. Empowering communities in combating river blindness and the role of NGOs: case studies from Cameroon, Mali, Nigeria, and Uganda. Health Res Policy Sys. 2012;10:16. 10.1186/1478-4505-10-16 PMC3453498 22574885

[10] Benton B. Taming the lion's stare. Baltimore: John Hopkins University Press; 2020.

[11] World Health Organization . Elimination of human onchocerciasis: progress report 2020. Wkly Epidemiol Rec. 2021;96 (46):557–67.

[12] World Health Organization . Summary of global update on implementation of preventive chemotherapy against neglected tropical diseases in 2019. Wkly Epidemiol Rec. 2020;95(39):469–74.

[13] Amazigo UV, Leak SGA, Zoure HGM et al. Community-directed distributors—the “foot soldiers” in the fight to control and eliminate neglected tropical diseases. PLoS Negl Trop Dis. 2021;15(3):e0009088. 33661903 10.1371/journal.pntd.0009088 PMC7932156

[14] Hodgkin D, Jennisken F. Final report on the Mectizan donation programme evaluation: 25 years of Mectizan donation. Amsterdam: Hodgkin Advice and Facilitation; 2012.

[15] Republic of Liberia Ministry of Health . Master plan for neglected tropical diseases 2016–2020. Available from: https://espen.afro.who.int/system/files/content/resources/Partners%20matrix_08Nov2018_LBR.pdf [accessed 29 April 2023].

[16] World Bank . Population, total – Liberia (2019). Available from: https://data.worldbank.org/indicator/SP.POP.TOTL?locations=LR [accessed 28 September 2022].

[17] PopulationStat . Monrovia, Liberia: Population (2019). Available from: https://populationstat.com/liberia/monrovia [accessed 14 April 2023].

[18] United Nations Office for the Coordination of Humanitarian Affairs . Liberia – settlements (2017). Available from: https://data.humdata.org/dataset/liberia-settlements-0 [accessed 6 June 2021].

[19] Standby Task Force . Health Facilities in Guinea, Liberia, Mali and Sierra Leone (2014). Available from: https://data.humdata.org/dataset/health-facilities-in-guinea-liberia-mali-and-sierra-leone [accessed 6 June 2021].

[20] Humanitarian OpenStreetMap Team . Liberia populated places (OpenStreetMap export) (2021). Available from: https://data.humdata.org/dataset/hotosm_lbr_populated_places [accessed 6 June 2021].

[21] WorldPop Hub . Global High Resolution Population Denominators Project – funded by the Bill and Melinda Gates Foundation (OPP1134076). Available from: 10.5258/SOTON/WP00675 [accessed 6 June 2021].

[22] Ray N, Ebener S. AccessMod 3.0: computing geographic coverage and accessibility to health care services using anisotropic movement of patients. Int J Health Geogr. 2008;7:63. 19087277 10.1186/1476-072X-7-63 PMC2651127

[23] World Health Organization . AccessMod: Supporting Universal Health Coverage by Modelling Physical Accessibility to Health Care. Version 5.0. Geneva: World Health Organization; 2017.

[24] Karra K, Kontgis C, Statman-Weil Z et al. Global land use/land cover with Sentinel-2 and deep learning. 2021 IEEE International Geoscience and Remote Sensing Symposium IGARSS, Brussels, Belgium, 2021, pp. 4704–7. Available from: https://www.arcgis.com/apps/instant/media/index.html?appid=fc92d38533d440078f17678ebc20e8e2 [accessed 6 June 2021].

[25] United Nations Office for the Coordination of Humanitarian Affairs . Liberia – Roads (2018). Available from: https://data.humdata.org/dataset/liberia-roads [accessed 6 June 2021].

[26] Humanitarian OpenStreetMap Team . Liberia Waterways (OpenStreetMap Export) (2021). Available from: https://data.humdata.org/dataset/hotosm_lbr_waterways [accessed 6 June 2021].

[27] Jarvis A, Reuter HI, Nelson A et al. Hole-filled SRTM for the Globe Version 4, from the CGIAR-CSI SRTM 90m Database. 2008. Available from: https://cgiarcsi.community/data/srtm-90m-digital-elevation-database-v4-1/ [accessed 6 June 2021].

[28] Tobler W. Three presentations on geographical analysis and modelling. Technical Report 93-1. National Center for Geographic Information and Analysis, University of California, Santa Barbara; 1993.

[29] Chen YN, Schmitz MM, Serbanescu F et al. Geographic access modeling of emergency obstetric and neonatal care in Kigoma Region, Tanzania: transportation schemes and programmatic implications. Glob Health Sci Pract. 2017;5(3):430–45. 28839113 10.9745/GHSP-D-17-00110 PMC5620339

[30] Wood SN. Generalized additive models: an introduction with R. New York: Chapman & Hall/CRC; 2017.

[31] Marra G, Wood SN. Practical variable selection for generalized additive models. Comput Stat Data Anal. 2011;55(7):2372–87.

[32] Locational //spatial intelligence . Spatial prediction. Available from: https://www.locational.io/docs/use-cases/spatial-prediction.html [accessed 12 November 2021].

[33] Zollner C, De Allegi M, Louis VR et al. Insecticide-treated mosquito nets in rural Burkina Faso: Assessment of coverage and equity in the wake of a universal distribution campaign. Health Policy Plan. 2015;30 (2):171–80. 24463333 10.1093/heapol/czt108

[34] World Bank . Better health in Africa; experience and lessons learned. Washington, DC: World Bank; 1994.

[35] Keenan JD, Bailey RL, West SK et al. Azithromycin to reduce childhood mortality in Sub-Saharan Africa. N Engl J Med. 2018;378(17):1583–92. 29694816 10.1056/NEJMoa1715474 PMC5849140

[36] Molyneux D, Bush S, Bannerman R et al. Neglected tropical diseases activities in Africa in the COVID‑19 era: the need for a “hybrid” approach in COVID‑endemic times. Infect Dis Poverty. 2021;10:1. 33397494 10.1186/s40249-020-00791-3 PMC7779653

[37] Adams AM, Vuckovic M, Birch E et al. Eliminating neglected tropical diseases in urban areas: a review of challenges, strategies and research directions for successful mass drug administration. Trop Med Infect Dis. 2018;3(4):122. 30469342 10.3390/tropicalmed3040122 PMC6306919

[38] African Development Bank Group . Tracking Africa's progress in figures. Tunis: African Development Bank; 2014. Available from: https://www.afdb.org/fileadmin/uploads/afdb/Documents/Publications/Tracking_Africa's_Progress_in_Figures.pdf [accessed 29 April 2023].

